# Early growth hormone treatment start in childhood growth hormone deficiency improves near adult height: analysis from NordiNet® International Outcome Study

**DOI:** 10.1530/EJE-16-1024

**Published:** 2017-08-03

**Authors:** Michel Polak, Jo Blair, Primoz Kotnik, Effie Pournara, Birgitte Tønnes Pedersen, Tilman R Rohrer

**Affiliations:** 1 Endocrinologie Gynécologie Diabétologie PédiatriquesHôpital Universitaire Necker Enfants Malades, Assistance Publique-Hôpitaux de Paris Université Paris Descartes, INSERM U1016, Institut IMAGINE, Centre de Référence des Maladies Endocriniennes Rares de la Croissance, Paris, France; 2 Department of EndocrinologyAlder Hey Children’s NHS Foundation Trust, Liverpool, UK; 3 Department of Pediatric EndocrinologyUniversity Children’s Hospital, University Medical Center Ljubljana, and Medical Faculty, University of Ljubljana, Ljubljana, Slovenia; 4 Global Medical AffairsNovo Nordisk Health Care AG, Zürich, Switzerland; 5 EpidemiologyNovo Nordisk A/S, Søborg, Denmark; 6 Department of Pediatric EndocrinologyUniversity Children’s Hospital, Saarland University Medical Center, Homburg, Germany

## Abstract

**Objective:**

To investigate the effect of age at growth hormone (GH) treatment start on near adult height (NAH) in children with isolated GH deficiency (GHD).

**Design:**

NordiNet® International Outcome Study (IOS) (Nbib960128), a non-interventional, multicentre study, evaluates the long-term effectiveness and safety of Norditropin® (somatropin) (Novo Nordisk A/S) in the real-life clinical setting.

**Methods:**

Patients (*n* = 172) treated to NAH (height at ≥18 years, or height velocity <2 cm/year at ≥16 (boys) or ≥15 (girls) years) were grouped by age (years) at treatment start (early (girls, <8; boys, <9), intermediate (girls, 8–10; boys, 9–11) or late (girls, >10; boys, >11)) and GHD severity (<3 ng/mL or 3 to ≤10 ng/mL). Multiple regression analysis was used to evaluate the effect of age at treatment start (as a categorical and continuous variable) on NAH standard deviation score (SDS).

**Results:**

Age at treatment start had a marked effect on NAH SDS; NAH SDS achieved by patients starting treatment early (*n* = 40 (boys, 70.0%); least squares mean (standard error) −0.76 (0.14)) exceeded that achieved by those starting later (intermediate, *n* = 42 (boys, 57.1%); −1.14 (0.15); late, *n* = 90 (boys, 68.9%); −1.21 (0.10)). Multiple regression analysis showed a significant association between NAH SDS and age at treatment start (*P* < 0.0242), baseline height SDS (HSDS) (*P* < 0.0001), target HSDS (*P* < 0.0001), and GHD severity (*P* = 0.0012). Most (78.5%) patients achieved a normal NAH irrespective of age at treatment start.

**Conclusions:**

Early initiation of GH treatment in children with isolated GHD improves their chance of achieving their genetic height potential.

## Introduction

Treatment with growth hormone (GH) is approved for children with GH deficiency (GHD) to normalise height during childhood and enable the achievement of an adult height within the normal range of the general population (1). Once a diagnosis of GHD has been made it is recommended that treatment with GH is initiated as soon as possible (1).

Data from some studies reported before 2000 suggested that although GH treatment was successful in increasing adult height, many patients with GHD were not achieving their full height potential (2, 3). However, more recent data suggest that a greater proportion of GH-treated patients with GHD are currently achieving an adult height within the normal range than reported in earlier studies (4, 5). In particular, an analysis from the Pfizer International Growth Study Database (KIGS) of the effect of GH treatment (years of treatment: girls, ≤11.7 years; boys, ≤12.1 years) on final height outcomes in children with idiopathic GHD showed that it is possible to achieve an adult height within the mid-parental height range (4). The data revealed a strong correlation between the prepubertal height increment and total height gain (4). In a later report on data from the same large multinational registry, 89% of GH-treated patients with isolated GHD and 81% of those with multiple pituitary hormone deficiency achieved a near adult height (NAH) within their genetic potential, with most of the height gain associated with GH therapy occurring in prepubertal years (5). More recently, Ross *et al.* reported that among a cohort of older patients (mean (standard deviation (s.d.)) age at GH treatment start 14.0 (2.1) years) with delayed skeletal maturation enrolled in the American Norditropin® Studies: Web-Enabled Research (ANSWER) Program, the majority of GH-treated patients, including those with idiopathic GHD, attained an NAH standard deviation score (SDS) within the normal range (6).

Growth prediction models based on accumulated data on the GH treatment response in prepubertal children with GHD enrolled in KIGS showed that severity of GHD was the most important predictor of the first-year response to GH treatment and height velocity during the previous year, body weight SDS and GH dose (all positively correlated) and chronological age (negatively correlated) were strong predictors of the growth response during the second, third and fourth years (7). In a study developing models for prediction of adult height in GH-treated children with GHD, height SDS (HSDS) at start or after the first year of GH treatment and target HSDS were found to be the most important predictive factors (8). Furthermore, the negative coefficient of bone age on adult height in the prediction model for prepubertal children was considered to reflect that start of GH treatment at a younger age results in a higher growth response (8).

In the present report, we evaluated the effect of age at GH treatment start on NAH SDS in children with isolated GHD enrolled in the NordiNet® International Outcome Study (IOS; Nbib960128). We also assessed the effect of HSDS at treatment start, mean GH dose during treatment, target (mid-parental) HSDS and the severity of GHD on NAH SDS.

## Subjects and methods

### Subjects and study design

The NordiNet® International Outcome Study (IOS) is a non-interventional, multicentre study evaluating the long-term effectiveness and safety of Norditropin® (somatropin) (Novo Nordisk A/S) as prescribed by the treating physicians in the real-life clinical setting. NordiNet® IOS was launched in 2006 and data are collected from 23 countries (9). All patients and/or their parents or caregivers gave informed consent prior to study enrolment and could withdraw from the study at any time. The study was conducted in accordance with the Declaration of Helsinki and was approved by the local Institutional Ethics Committee/Institutional Review Board and the local regulatory authorities at each study centre and data privacy agencies as required. NordiNet® IOS is conducted in accordance with the Good Pharmacoepidemiology Practice guidelines (10).

Patients enrolled in NordiNet® IOS up to July 2016 who were diagnosed with isolated GHD by their treating physician, had a GH stimulation test peak value ≤10 ng/mL and had achieved NAH, were included in the present analysis. NAH was defined as the height achieved at ≥18 years of age, if height velocity was <2 cm per year at age ≥16 years (boys) or ≥15 years (girls). A sensitivity analysis was performed on all patients diagnosed with isolated GHD by their treating physician and who had reached NAH, irrespective of whether they had recorded data on a GH stimulation test.

Patients were classified according to age at GH treatment start: early (girls aged <8 years; boys aged <9 years), intermediate (girls aged 8–10 years; boys aged 9–11 years) or late age group (girls aged >10 years; boys aged >11 years). Furthermore, patients were categorised according to the severity of GHD: severe GHD (GH stimulation peak <3 ng/mL) and non-severe GHD group (GH stimulation peak 3 to ≤10 ng/mL). Target (mid-parental) height was calculated by adding 6.5 cm to the mean of the parents’ heights for boys or by subtracting 6.5 cm from the mean of the parents’ heights for girls (11). Onset of puberty was defined as Tanner breast stage ≥2 in girls and testicular volume ≥4 mL in boys.

NAH SDS and change in HSDS from baseline to NAH (∆HSDS) were assessed. HSDS was calculated using corresponding national reference standards.

### Statistical analysis

Descriptive statistics were applied on all parameters and are presented as mean (s.d.) or median (range). Differences in baseline parameters between the respective age and severity of GHD groups were calculated and evaluated by *t*-test statistics. The effect of age on NAH SDS and ∆HSDS was evaluated by a multiple regression model, which included additional parameters possibly associated with height outcome to adjust for potential bias. The model included HSDS at GH treatment start, mean GH dose during treatment, target HSDS and severity of GHD. Age and GH test peak value were analysed as categorical variables; all other parameters were analysed as continuous variables. NAH SDS and ∆HSDS were also analysed using a model with age at treatment start included as a continuous variable. Missing data were considered missing completely at random. Estimated NAH SDS and ∆HSDS are presented as least squares (LS) means and standard error (s.e.). As information on GH stimulation test results was not always available in the study database, a sensitivity analysis was performed applying the same models on a dataset including all children diagnosed with isolated GHD who had reached NAH, without including the GH test peak value as an explanatory variable. Statistical analysis was performed using SAS v9.4 (SAS Institute Inc., Cary, NC, USA).

## Results

### Study cohort and patient characteristics

Overall, 16 668 paediatric patients were enrolled in the study up to July 2016. Of these, 9294 patients were diagnosed with GHD, 8166 with isolated GHD and 1128 with multiple pituitary hormone deficiency. As patients were enrolled continuously throughout the study period, only 350 (4.3%) of the 8166 patients diagnosed with isolated GHD achieved NAH within the study period, of whom 172 (2.1%) had a GH stimulation test peak value ≤10 ng/mL. The 172 patients (66.3% male) were included in this analysis. Patient characteristics are presented in Table 1 by age at treatment start, and in Table 2 by age at treatment start and severity of GHD. Median (range) age at treatment start was 10.8 (2.5–16.5) years: 52.3% of patients were included in the late age group (mean (s.d.) age at treatment start, 12.3 (1.4) years), 24.4% in the intermediate (9.7 (0.9) years) and 23.3% in the early (6.4 (1.8) years) age groups. Bone age was slightly retarded compared with chronological age at treatment start in all three age groups; however, information on bone age was only available in 48 patients (Table 1).

**Table 1:** Characteristics of study population by age group. Data are mean (s.d.) unless otherwise stated.

	**Age group**		
	Early (***n* = 40**)	Intermediate (***n* = 42**)	Late (***n* = 90**)
Age at GH treatment start (years)	6.4 (1.8)	9.7 (0.9)	12.3 (1.4)
Male, *n* (%)	28 (70.0)	24 (57.1)	62 (68.9)
Age at puberty (years)	12.4 (1.5)^a^	12.2 (1.2)^b^	13.5 (1.6)^c^
Prepubertal treatment period (years)	6.0 (2.6)^d^	2.4 (1.0)^e^	1.3 (1.1)^f^
HSDS at baseline	−3.2 (0.9)	−2.7 (0.8)	−2.8 (1.0)
Bone age at start of treatment (years)	5.3 (2.5)^g^	8.1 (1.0)^h^	10.0 (1.8)^i^
Bone age/chronological age at start of treatment	0.8 (0.3)^g^	0.9 (0.1)^h^	0.8 (0.2)^i^
Target (mid-parental) HSDS	−0.8 (1.1)	−1.2 (0.9)^j^	−0.7 (0.8)^k^
Age at NAH (years)	16.6 (1.3)	16.4 (1.3)	17.4 (1.3)
Age at last presentation (years)	17.7 (1.7)	17.0 (1.3)	17.9 (1.2)
GH dose at baseline (µg/kg/day)	33.8 (13.2)^j^	28.6 (7.5)^l^	30.1 (8.3)^m^
Duration of GH treatment from baseline to NAH (years)	10.2 (2.1)	6.5 (1.2)	5.0 (0.8)
GH average dose during treatment period (μg/kg/day)	32.7 (5.6)	32.9 (7.0)^n^	31.6 (6.5)
NAH SDS	−1.0 (1.2)	−1.5 (0.9)	−1.3 (1.1)
Difference between NAH and target (mid-parental) height SDS	−0.2 (0.9)	−0.2 (1.0)^j^	−0.6 (1.2)^k^
∆HSDS	2.3 (1.3)	1.4 (0.9)	1.5 (0.9)
Mean GH stimulation test peak value (ng/mL)	4.6 (2.4)	5.7 (2.4)	4.8 (2.5)

Early (girls aged <8 years; boys aged <9 years), intermediate (girls aged 8–10 years; boys aged 9–11 years) or late (girls aged >10 years; boys aged >11 years).

a *n* = 19, ^b^*n* = 26, ^c^*n* = 50, ^d^*n* = 24, ^e^*n* = 29, ^f^*n* = 56, ^g^*n* = 11, ^h^*n* = 12, ^i^*n* = 25, ^j^*n* = 39, ^k^*n* = 86, ^l^*n* = 40, ^m^*n* = 83, ^n^*n* = 41.

GH, growth hormone; HSDS, height standard deviation score; ∆HSDS, change in height standard deviation score from baseline to NAH; NAH, near adult height; s.d., standard deviation; SDS, standard deviation score.

**Table 2:** Characteristics of study population by age group and by severity of GHD (GH stimulation test peak value: severe GHD: <3 ng/mL or non-severe GHD: 3 to ≤10 ng/mL). Data are mean (s.d.) unless otherwise stated.

	**Age group**					
	Early (***n* = 40**)		Intermediate (***n* = 42**)		Late (***n* = 90**)	
	Severe GHD (***n* =** **12**)	Non-severe GHD (*n* = **28**)	Severe GHD (***n* = 7**)	Non-severe GHD (***n* =** **35**)	Severe GHD (***n* = 22**)	Non-severe GHD (***n* =** **68**)
Age at GH treatment start (years)	5.2 (2.0)	6.8 (1.5)	9.4 (0.8)	9.8 (0.9)	12.8 (1.9)	12.1 (1.1)
Male, *n* (%)	9 (22.5)	19 (47.5)	3 (7.1)	21 (50.0)	16 (17.8)	46 (51.1)
Age at puberty (years)	12.9 (2.6)^a^	12.3 (1.3)^b^	11.9 (0.1)^a^	12.3 (1.3)^c^	14.0 (2.2)^b^	13.3 (1.2)^d^
Prepubertal treatment period (years)	9.2 (3.0)^e^	5.0 (1.4)^f^	2.5 (1.1)^a^	2.4 (1.0)^g^	1.1 (1.0)^f^	1.5 (1.1)^h^
HSDS at baseline	−3.5 (1.1)	−3.0 (0.8)	−3.4 (1.1)	−2.6 (0.7)	−3.1 (1.2)	−2.7 (0.9)
Target (mid-parental) HSDS	−0.2 (0.7)	−1.0 (1.1)	−0.6 (1.2)	−1.3 (0.8)	−0.6 (0.7)^i^	−0.7 (0.9)^j^
Age at NAH (years)	17.0 (1.6)	16.5 (1.1)	16.9 (0.6)	16.2 (1.4)	17.7 (1.7)	17.2 (1.2)
GH dose at baseline (μg/kg/day)	40.3 (20.5)^k^	31.3 (8.2)	26.8 (7.6)	29.0 (7.5)^l^	30.1 (11.7)^i^	30.1 (7.0)^m^
Duration of GH treatment from baseline to NAH (years)	11.7 (2.6)	9.5 (1.5)	7.3 (1.5)	6.3 (1.1)	4.9 (0.8)	5.1 (0.8)
GH average dose during treatment period (μg/kg/day)	33.0 (5.5)	32.5 (5.8)	28.8 (4.5)	33.7 (7.1)^d^	30.9 (5.6)	31.9 (6.8)
NAH SDS	−0.1 (1.0)	−1.4 (1.0)	−0.8 (1.1)	−1.6 (0.8)	−1.4 (1.2)	−1.2 (1.0)
Difference between NAH and target (mid-parental) height SDS	0.1 (0.7)	−0.4 (0.9)	−0.2 (0.5)	−0.2 (1.0)^n^	−0.7 (1.1)^i^	−0.5 (1.3)^j^
∆HSDS	3.5 (1.3)	1.7 (1.0)	2.7 (1.3)	1.1 (0.6)	1.8 (1.3)	1.5 (0.8)
Mean GH stimulation test peak value (ng/mL)	1.7 (0.8)	5.8 (1.8)	1.8 (0.7)	6.5 (1.8)	1.5 (0.9)	5.9 (1.8)

a *n* = 3, ^b^*n* = 16, ^c^*n* = 23, ^d^*n* = 34, ^e^*n* = 6, ^f^*n* = 18, ^g^*n* = 26, ^h^*n* = 38, ^i^*n* = 20, ^j^*n* = 66, ^k^*n* = 11, ^l^*n* = 33, ^m^*n* = 63, ^n^*n* = 32.

GH, growth hormone; HSDS, height standard deviation score; ∆HSDS, change in height standard deviation score from baseline to NAH; NAH, near adult height; s.d., standard deviation; SDS, standard deviation score.

Almost one-quarter (23.8%) of patients had severe GHD; of these, 53.7, 29.2 and 17.1% were in the late, early and intermediate age groups, respectively (Table 2). Based on the information available in the database, the most commonly used test was arginine, which represented 29.9% (*n* = 93) of tests applied, followed by the insulin tolerance test (ITT), representing 19.0% (*n* = 59); clonidine, representing 16.4% (*n* = 51); arginine + ITT, representing 8.7% (*n* = 27) of tests applied; and various other tests, representing 26% (*n* = 81) of the tests applied. More than one type of test could have been used for an individual patient.

There were more boys than girls in each age group, with a greater proportion of boys in the early and late age groups than in the intermediate age group (Table 1). HSDS was lower before GH treatment start in children in the early age group compared with those in the intermediate (mean difference (95% confidence interval; CI), −0.41 (−0.83; 0.00); *P* = 0.0508) or late (mean difference (95% CI), −0.37 (−0.73; −0.02); *P* = 0.0399) age group (Table 1). Furthermore, at baseline, patients with severe GHD had lower mean HSDS than those with non-severe GHD across all age groups (Table 2).

Target HSDS was lower for patients in the intermediate age group than for patients in the early age group (mean difference (95% CI), −0.42 (−0.01; −0.82); *P* = 0.0432) or late age group (−0.52 (−0.87; −0.18); *P* = 0.003).

### Effect of GH replacement

#### NAH SDS

As expected, due to the earlier initiation of GH treatment, the mean duration of treatment to NAH was significantly longer for patients starting treatment at a young age than for older patients (*P* < 0.0001 early vs both intermediate and late groups; Table 1), with patients in the early age group receiving GH treatment for almost twice as long as patients in either of the other two groups. Overall, treatment duration was significantly longer for patients with severe GHD than for those with non-severe GHD in the early (*P* < 0.0001) and intermediate (*P* = 0.0408) age groups (Table 2), but was similar between those with severe GHD and non-severe GHD in the late age group.

Patients in the early age group who started treatment at a young age achieved a greater mean (s.d.) NAH SDS (−1.0 (1.2)) than those who were older at treatment start (intermediate, −1.5 (0.9); late, −1.3 (1.1) age groups; Table 1). NAH SDS was observed to differ by severity of GHD among patients in the early and intermediate age groups only, being higher for patients with severe GHD than for those with non-severe GHD (Table 2). The majority (78.5%) of all patients achieved a normal NAH >−2 SDS: 77.5, 73.8 and 81.1% in the early, intermediate and late age groups, respectively.

**Figure 1:**
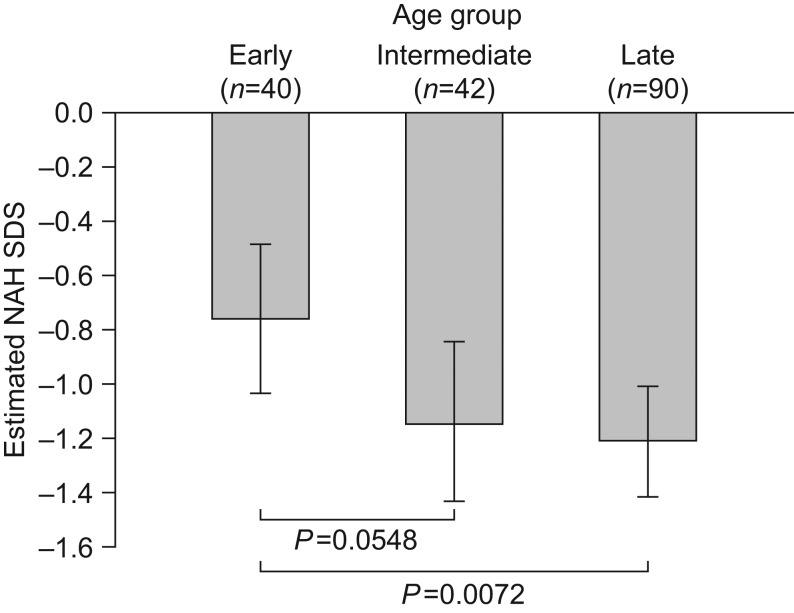
Estimated NAH SDS by early (girls aged <8 years; boys aged <9 years), intermediate (girls aged 8–10 years; boys aged 9–11 years) and late (girls aged >10 years; boys aged >11 years) age group at GH treatment start. Data are LS means (95% CI) corrected for baseline HSDS, average GH dose, target HSDS, GH severity and mid-parental height. CI, confidence interval; GH, growth hormone; HSDS, height standard deviation score; LS, least squares; NAH, near adult height; SDS, standard deviation score.

Multiple linear regression analyses were used to adjust for additional effect modifiers when evaluating the effect of age at treatment start on NAH SDS. Age was included in the model as a categorical variable and was shown to be a significant predictor of NAH SDS (*P* = 0.0242); the estimated mean NAH SDS (LS mean (s.e.)) was −0.76 (0.14) in the early, −1.14 (0.15) in the intermediate and −1.21 (0.10) in the late age group. Hence, a significant difference in NAH SDS was found between patients starting treatment early and those starting treatment late (estimated mean difference, 0.45 SDS; *P* = 0.0072), indicating a better treatment outcome in the early age group. An estimated mean difference in NAH SDS of 0.38 (*P* = 0.0548) was observed between patients in the early and intermediate age groups and an estimated mean difference of 0.07 (*P* = 0.6889) was observed between the intermediate and late age groups (Fig. 1). Incorporating age as a continuous variable into the model confirmed the significant effect of age at treatment start on NAH SDS (estimated effect (EE) = −0.06; *P* = 0.0114) (Table 3). In addition to age at treatment start, significant predictive factors of NAH SDS were baseline HSDS, severity of GHD and target HSDS, but not GH dose (Table 3). A strong positive association was shown between NAH SDS and baseline HSDS and target HSDS, and a negative association was found between NAH SDS and GH stimulation test peak value. NAH SDS was statistically significantly greater for patients with severe GHD than for those with non-severe GHD.

**Table 3:** Estimated effects and *P* values from the multiple regression analysis* examining the effect of different parameters on NAH SDS and change in HSDS from baseline to NAH SDS.

	**NAH SDS**		**∆HSDS**	
**Parameter**	**Estimated effect** (s.e.)	***P* value**	**Estimated effect** (s.e.)	***P* value**
Age as a categorical variable				
Baseline HSDS	0.49 (0.07)	<0.0001	−0.48 (0.07)	<0.0001
GH dose (μg/kg/day)	−0.02 (0.01)	0.1119	−0.02 (0.01)	0.0773
GHD severity^†^	0.55 (0.17)	0.0012	0.57 (0.16)	0.0005
Target (mid-parental) HSDS	0.39 (0.08)	<0.0001	0.36 (0.07)	<0.0001
Age group				
Early vs intermediate	0.38 (0.20)	0.0548	0.40 (0.19)	0.0358
Early vs late	0.45 (0.16)	0.0072	0.54 (0.16)	0.0010
Intermediate vs late	0.07 (0.17)	0.6889	0.13 (0.17)	0.4279
Age as a continuous variable				
Age at treatment start	−0.06 (0.02)	0.0114	−0.08 (0.02)	0.0010
Baseline HSDS	0.48 (0.07)	<0.0001	−0.49 (0.07)	<0.0001
GH dose (μg/kg/day)	−0.02 (0.01)	0.0857	−0.02 (0.01)	0.0542
GHD severity^†^	0.54 (0.17)	0.0015	0.56 (0.16)	0.0007
Target (mid-parental) HSDS	0.39 (0.07)	<0.0001	0.36 (0.07)	<0.0001

* Adjusted for baseline age, baseline HSDS, GH dose, GH stimulation test peak value, and target (mid-parental) HSDS.

† Patients with severe GHD (peak value <3 ng/mL) were compared with patients with non-severe GHD (peak value 3 to ≤10 ng/mL) as reference.

GH, growth hormone; GHD, growth hormone deficiency; HSDS, height standard deviation score; ∆HSDS, change in height standard deviation score from baseline to NAH; NAH, near adult height; SDS, standard deviation score; s.e., standard error.

#### ∆HSDS

**Figure 2:**
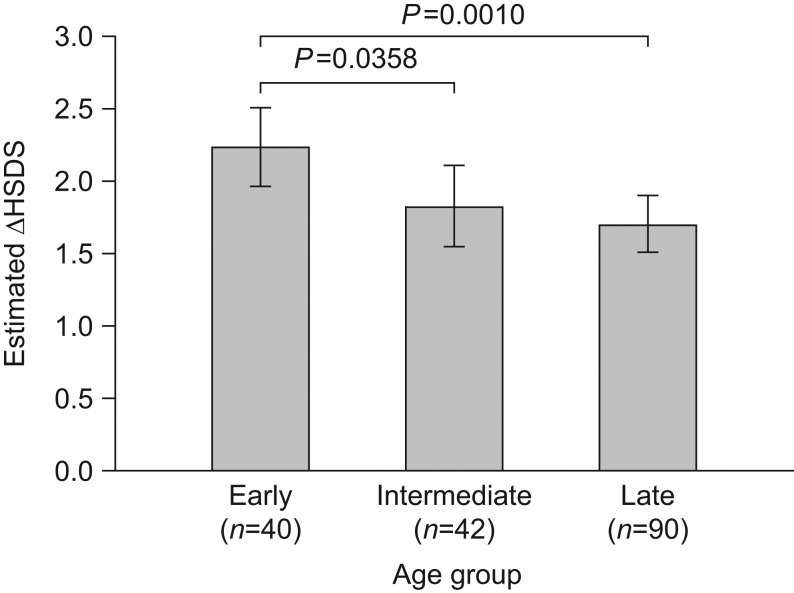
Estimated change in HSDS from baseline to NAH by early (girls aged <8 years; boys aged <9 years), intermediate (girls aged 8–10 years; boys aged 9–11 years) and late (girls aged >10 years; boys aged >11 years) age group at GH treatment start. Data are LS means (95% CI) corrected for baseline HSDS, average GH dose, target HSDS, GH severity and mid-parental height. CI, confidence interval; GH, growth hormone; HSDS, height standard deviation score; ΔHSDS, change in height standard deviation score from baseline to NAH; LS, least squares; NAH, near adult height.

As observed for NAH SDS, overall mean (s.d.) ∆HSDS was greater in patients who started treatment early (2.3 (1.3)) than in those starting treatment later (intermediate, 1.4 (0.9); late, 1.5 (0.9)). Applying the same model used to analyse NAH SDS, with age and GH test peak value as categorical variables, age at treatment start was also found to be a significant predictor of ∆HSDS (*P* = 0.0044); the estimated mean ∆HSDS (LS mean (s.e.)) was 2.22 (0.14) in the early, 1.82 (0.14) in the intermediate and 1.69 (0.10) in the late age group. ∆HSDS was significantly greater for patients in the early age group than for those in the intermediate age group (between-group difference, 0.40; *P* = 0.0358) and late age group (between-group difference, 0.54; *P* = 0.0010); a non-significant mean difference of 0.13 (*P* = 0.4279) was shown between patients in the intermediate and late age groups (Fig. 2 and Table 2). The significant effect of age at treatment start on ∆HSDS was confirmed when the analysis was repeated including age as a continuous variable in the model (EE = −0.08; *P* = 0.001). Findings for other significant predictors of ∆HSDS were consistent with those for NAH SDS including severity of GHD (*P* = 0.0005); however, in contrast with NAH SDS, baseline HSDS was inversely associated with ∆HSDS (*P* < 0.0001) and target HSDS showed a strong positive association with ∆HSDS (*P* < 0.0001; Table 3). No significant effect of GH dose on ∆HSDS was observed (*P* = 0.0773).

### Sensitivity analysis

A sensitivity analysis performed for all children diagnosed with isolated GHD irrespective of availability of data from a GH stimulation test (*n* = 350) showed similar associations for NAH SDS and ∆HSDS to those presented for the patients with a confirmed diagnosis by GH stimulation test (data not shown).

#### GH dose

Younger patients started treatment on a significantly higher mean (s.d.) GH dose (33.8 (13.2) µg/kg/day) than those who were older on starting treatment (intermediate age group, 28.6 (7.5) µg/kg/day; *P* = 0.0161; late age group, 30.1 (8.3) µg/kg/day; *P* = 0.0478; Table 1). Furthermore, in the youngest patients, those with severe GHD received a significantly higher GH dose at baseline than those with non-severe GHD (*P* = 0.0076; Table 2). However, the median (range) average GH dose during the treatment period was similar between age groups (32.7 (20.5–44.5), 32.1 (21.3–49.1) and 32.1 (19.6–56.3) µg/kg/day in the early, intermediate and late age groups, respectively; Table 1). Moreover, although the average GH dose during the treatment period was similar between patients with severe GHD vs those with non-severe GHD in the early and late age groups (Table 2), a lower average GH dose was observed for patients with severe GHD vs those with non-severe GHD in the intermediate age group (*P* = 0.0682).

#### Puberty

Data on pubertal status were available for 125 patients. Of these, the majority of patients (88%) were prepubertal at baseline. Fifteen patients were pubertal at baseline; of these, 14 were in the late age group and one was in the intermediate age group. Patients who started treatment early were significantly younger at onset of puberty than those who started treatment later (early vs late age groups; *P* = 0.0063; Table 1). Likewise, patients in the intermediate age group were significantly younger at pubertal onset than those in the late age group (*P* = 0.0005; Table 1). Age at start of puberty was similar within age groups for patients with severe GHD and non-severe GHD (Table 2).

As a consequence of the earlier start of GH treatment, and despite their younger age at pubertal onset, the mean prepubertal treatment period was significantly longer for patients who started treatment early than for those starting later in the intermediate (*P* < 0.0001) or late age groups (*P* < 0.0001). The prepubertal treatment period was also significantly longer for patients in the intermediate vs the late age group (*P* = 0.0046; Table 1) and for patients with severe GHD vs those with non-severe GHD in the early age group (*P* < 0.0001; Table 2).

## Discussion

The results of the present analysis show that in children with isolated GHD, younger age at treatment start was associated with improved NAH SDS and greater ∆HSDS compared with older age at treatment start. Nonetheless, irrespective of age at treatment start, over three-quarters of all patients achieved a NAH SDS within the normal range (from −2 to 2), albeit at the lower end of the range for those who were older at treatment start.

The lower NAH SDS among patients who started treatment late compared with those who started treatment early may reflect their significantly shorter duration of prepubertal treatment despite their older age at pubertal onset. It is also possible that a proportion of children in the late age group had constitutional delay in growth and puberty (CDGP). Although CDGP can result in a longer period of growth during the prepubertal period (12), allowing for a degree of catch-up of growth, the gain in growth may not be sufficient to compensate for the height deficit at the start of puberty and the late start of GH replacement in these patients. However, as mean bone age delay was modest across all age groups, including those in the late age group, CDGP seems less likely to account for the short stature; bone age information was only available in a limited number of patients included in the study.

The differences in both NAH SDS and ∆HSDS were less pronounced between patients in the intermediate and the late age groups than between patients in the early and late age groups. A possible explanation for at least part of this difference is the aetiology of short stature in the different age groups. Although almost all of the patients in the early age group were likely to have had a definite diagnosis of GHD (biochemical deficiency in GH) it is possible that a greater proportion of the patients in the intermediate age group than in the late age group may have had a definite diagnosis of GHD and thus responded well to GH replacement whereas, as previously discussed, a proportion of those who started treatment late may have had CDGP. This study reports data on patients diagnosed with isolated GHD who achieved NAH and had a GH stimulation test peak value ≤10 ng/mL. The sensitivity analysis included all children diagnosed with isolated GHD who had reached NAH, regardless of whether a GH stimulation test peak value was recorded, and it cannot be ruled out that some of these patients may have been assigned a diagnosis to fit agreed prescribing indications.

A number of studies have reported that NAH or adult height outcomes in GH-treated children with idiopathic GHD are improved when treatment is started early (4, 5, 6, 8, 13). Reiter *et al*. reported strong correlations between total height gain, and the first-year increase in HSDS and prepubertal height gain in GH-treated children with idiopathic GHD, confirming the importance of starting treatment before pubertal onset (4). Another paper published a year later also reported change in HSDS during the first year of GH replacement as an important predictor of adult height in a model derived from a retrospective analysis of data from the National Registry of Growth Hormone Treatment in Dutch children with GHD (8). Data from 401 children with idiopathic GHD enrolled in the Swedish cohort of the KIGS study showed that early treatment start is associated with improved adult height outcomes; however, all children, irrespective of age at treatment start, achieved a final height within their genetic potential (13). A subsequent publication from the multinational KIGS data reported that 89% of GH-treated patients with isolated GHD reached a NAH within their genetic potential with most of the height gain occurring during the prepubertal years (5). Finally, a recent publication on data from the ANSWER Program, a significant negative association between age at GH treatment start and NAH SDS was observed in a cohort of older children with isolated GHD and delayed skeletal maturation (6).

Consistent with data from KIGS, we found that baseline HSDS and target HSDS, but not GH dose, were predictors of NAH SDS (5). Hence, patients with a higher target HSDS grew more than those with a lower target HSDS, which is not surprising given that short children with tall parents have a greater genetic height potential than those with short parents.

A statistically significant effect of severity of GHD on NAH SDS was observed in our study; patients with non-severe GHD demonstrated lower NAH SDS and lower ∆HSDS than those with severe GHD. This difference in outcomes, based on GHD severity, was most marked among children who were young at treatment start, which may suggest that the children in the intermediate and late age groups classified with severe GHD could have been misclassified. There is much debate over the reliability of GH stimulation tests and their ability to segregate patients with severe and non-severe GHD. Carel *et al.* (14) reported on the reproducibility of GH stimulation tests according to severity of GHD. Reliability of the tests was shown to be good for very low (<5 ng/mL) GH values, but decreased with increasing (5–10 ng/mL) GH values. Rosenfeld *et al.* (15) suggest diagnosis of GHD should be based on auxological and biochemical criteria, and that insulin-like growth factor-I and insulin-like growth factor-binding protein-3 may provide a more accurate diagnosis of GHD severity than GH stimulation testing alone. It is recognised that there is an overlap in peak GH concentrations between children with GHD and those with normal growth and CDGP, making both the diagnosis of GHD and classification of severity challenging (1). Therefore, it is possible that the GHD severity classification of the children in this study report may not have been optimal as a consequence of the reliance on available data from GH provocative tests for defining severe GHD rather than clinical or auxological criteria. However, our findings are in line with data from KIGS, which similarly demonstrated a more pronounced increase in HSDS to adult height in children with severe GHD (GH stimulation peak <3.3 μg/L) who started treatment before the age of 6 years than in children of the same age with partial GHD (GH stimulation peak ≥3.3 μg/L) or those who started treatment at an older age (13). Our results highlight the importance of adequately communicating to children with non-severe GHD and their carers the possibility of a more modest outcome at the end of treatment.

In the present study, GH dose was not shown to be significantly associated with NAH; this has also been reported by others (5, 8, 16) and is likely to reflect clinical practice, in which GH dose is individualised based on clinical response. Hence, GH dose may be increased in non-responders, or a high starting dose may be used in very short children or in children with non-severe GHD. However, GH dose has been shown to affect short-term growth (7) as it has an effect on first-year height velocity (5) with a possible resulting effect on NAH.

Consistent with other GH registries, we found that more males than females with GHD were treated with GH in NordiNet® IOS. Data indicating a male predominance among GH-treated paediatric patients with GHD were first reported in 1990 (17) and the male predominance has persisted for over three decades (18). The exact sex ratio depends on the age at treatment initiation and varies among different countries but may be as high as 2:1 (18).

There are strengths and limitations to the present study, given its observational design. As data were collected from several sites, differences in diagnostic and treatment practices, and in reporting standards, leading to incomplete or poor reporting of treatment exposure and outcomes, may exist. Potential bias cannot be ruled out due to the enrolment of selected clinics and also due to the selection of a subset of patients with available GH stimulation test peak values. As the extent of the severity of GHD differed between the three age groups, with those in the early age group potentially having a more severe GHD than those in the intermediate and late age groups, this could possibly influence the results. Potential bias between age groups was adjusted for by including severity of GHD as a covariate in the model. However, the type of GH stimulation test used could have impacted the classification of GHD severity. Furthermore, because only patients who achieved NAH were included in the analysis and patient enrolment was continuous, our analysis (and the distribution of the data) is likely to be skewed towards those patients who were older at the time of treatment start, as those who were younger on starting GH treatment are less likely to have attained NAH at the time of this analysis unless they started GH treatment in the early years of the study. Finally, no data on adherence with treatment were available.

Major strengths of the present study include the long-term follow-up data to NAH that are captured in a large database, collected over a 10-year period, and reflecting real-world clinical practice across multiple countries; these data add new, contemporary evidence to previously published results. We therefore consider the present analysis to add weight and further expand on the evidence reported in the existing literature (4, 5, 6, 8, 13).

In conclusion, the data from the present study highlight that starting GH treatment early in children with isolated GHD is associated with improved NAH SDS compared with starting GH treatment later. Furthermore, these data being representative of real-life clinical practice may suggest that a proportion of children with isolated GHD start GH treatment late, even though compelling evidence shows that they would benefit most from starting GH treatment at an early age. Correcting the observed referral bias reflecting the predominance of boys will be important to avoid delayed or missed diagnoses in girls. Finally, the results of the study suggest that the severity of GHD is associated with the NAH achieved, but the influence of the GH stimulation test used for measuring GHD severity remains a matter of debate.

## Declaration of interest

M Polak, J Blair, P Kotnik and T Rohrer received payment for their role as a member of the International Steering Committee for NordiNet® IOS. M Polak is a member of the Global Norditropin® Advisory Panel (Novo Nordisk) and the board of the INCRELEX Registry (IPSEN). J Blair received honoraria from Novo Nordisk. P Kotnik received honoraria from Novo Nordisk. TR Rohrer received honoraria from Novo Nordisk. B Tønnes Pedersen is an employee of Novo Nordisk. E Pournara was an employee of Novo Nordisk at the time of this study.

## Funding

This project was sponsored by Novo Nordisk Health Care AG, Zürich, Switzerland.

## References

[1] GH Research Society. Consensus guidelines for the diagnosis and treatment of growth hormone (GH) deficiency in childhood and adolescence: summary statement of the GH research society. Journal of Clinical Endocrinology and Metabolism 2000 85 3990–3993. (10.1210/jcem.85.11.6984) 11095419

[2] Blethen SL Baptista J Kuntze J Foley T LaFranchi S Johanson A. Adult height in growth hormone (GH)-deficient children treated with biosynthetic GH. The Genentech Growth Study Group. Journal of Clinical Endocrinology and Metabolism 1997 82 418–420. (10.1210/jc.82.2.418) 9024229

[3] Cacciari E Cicognani A Pirazzoli P Zucchini S Salardi S Balsamo A Cassio A Pasini A Carla G Tassinari D Final height of patients treated for isolated GH deficiency: examination of 83 patients. European Journal of Endocrinology 1997 137 53–60. (10.1530/eje.0.1370053) 9242202

[4] Reiter EO Price DA Wilton P Albertsson-Wikland K Ranke MB. Effect of growth hormone (GH) treatment on the near-final height of 1258 patients with idiopathic GH deficiency: analysis of a large international database. Journal of Clinical Endocrinology and Metabolism 2006 91 2047–2054. (10.1210/jc.2005-2284) 16537676

[5] Darendeliler F Lindberg A Wilton P. Response to growth hormone treatment in isolated growth hormone deficiency versus multiple pituitary hormone deficiency. Hormone Research in Paediatrics 2011 76 (Supplement 1) 42–46. (10.1159/000329161) 21778748

[6] Ross JL Lee PA Gut R Germak J. Increased height standard deviation scores in response to growth hormone therapy to near-adult height in older children with delayed skeletal maturation: results from the ANSWER Program. International Journal of Pediatric Endocrinology 2015 2015 1 (10.1186/s13633-015-0015-1) 25904938 PMC4405836

[7] Ranke MB Lindberg A Chatelain P Wilton P Cutfield W Albertsson-Wikland K Price DA. Derivation and validation of a mathematical model for predicting the response to exogenous recombinant human growth hormone (GH) in prepubertal children with idiopathic GH deficiency. KIGS International Board. Kabi Pharmacia International Growth Study. Journal of Clinical Endocrinology and Metabolism 1999 84 1174–1183. (10.1210/jc.84.4.1174) 10199749

[8] de Ridder MA Stijnen T Hokken-Koelega AC. Prediction of adult height in growth-hormone-treated children with growth hormone deficiency. Journal of Clinical Endocrinology and Metabolism 2007 92 925–931. (10.1210/jc.2006-1259) 17179199

[9] Hoybye C Savendahl L Christesen HT Lee P Pedersen BT Schlumpf M Germak J Ross J The NordiNet(R) International Outcome Study and NovoNet(R) ANSWER Program(R): rationale, design, and methodology of two international pharmacoepidemiological registry-based studies monitoring long-term clinical and safety outcomes of growth hormone therapy (Norditropin(R)). Clinical Epidemiology 2013 5 119–127. (10.2147/CLEP.S42602) 23658497 PMC3641810

[10] Public Policy Committee ISoP. Guidelines for good pharmaco­epidemiology practice (GPP). Pharmacoepidemiology and Drug Safety 2016 25 2–10. (10.1002/pds.3891) 26537534

[11] Tanner JM Goldstein H Whitehouse RH. Standards for children’s height at ages 2–9 years allowing for heights of parents. Archives of Disease in Childhood 1970 45 755–762. (10.1136/adc.45.244.755) 5491878 PMC1647404

[12] Rogol AD Clark PA Roemmich JN. Growth and pubertal development in children and adolescents: effects of diet and physical activity. American Journal of Clinical Nutrition 2000 72 521s–528s. 10919954 10.1093/ajcn/72.2.521S

[13] Westphal O Lindberg A Final height in Swedish children with idiopathic growth hormone deficiency enrolled in KIGS treated optimally with growth hormone. Acta Paediatrica 2008 97 1698–1706. (10.1111/j.1651-2227.2008.01053.x) 18976357

[14] Carel JC Tresca JP Letrait M Chaussain JL Lebouc Y Job JC Coste J. Growth hormone testing for the diagnosis of growth hormone deficiency in childhood: a population register-based study. Journal of Clinical Endocrinology and Metabolism 1997 82 2117–2021. (10.1210/jc.82.7.2117) 9215281

[15] Rosenfeld RG Albertsson-Wikland K Cassorla F Frasier SD Hasegawa Y Hintz RL Lafranchi S Lippe B Loriaux L Melmed S Diagnostic controversy: the diagnosis of childhood growth hormone deficiency revisited. Journal of Clinical Endocrinology and Metabolism 1995 80 1532–1534. (10.1210/jc.80.5.1532) 7538145

[16] Carel JC Ecosse E Nicolino M Tauber M Leger J Cabrol S Bastie-Sigeac I Chaussain JL Coste J. Adult height after long term treatment with recombinant growth hormone for idiopathic isolated growth hormone deficiency: observational follow up study of the French population based registry. BMJ 2002 325 70–77. (10.1136/bmj.325.7355.70) 12114235 PMC117125

[17] August GP Lippe BM Blethen SL Rosenfeld RG Seelig SA Johanson AJ Compton PG Frane JW McClellan BH Sherman BM. Growth hormone treatment in the United States: demographic and diagnostic features of 2331 children. Journal of Pediatrics 1990 116 899–903. (10.1016/S0022-3476(05)80647-X) 2348293

[18] Grimberg A Stewart E Wajnrajch MP. Gender of pediatric recombinant human growth hormone recipients in the United States and globally. Journal of Clinical Endocrinology and Metabolism 2008 93 2050–2056. (10.1210/jc.2007-2617) 18334582 PMC2435638

